# Differential Effects of Focused Attention and Open Monitoring Meditation on Autonomic Cardiac Modulation and Cortisol Secretion

**DOI:** 10.3389/fphys.2021.675899

**Published:** 2021-07-15

**Authors:** Yuuki Ooishi, Masahiro Fujino, Vimala Inoue, Michio Nomura, Norimichi Kitagawa

**Affiliations:** ^1^NTT Communication Science Laboratories, NTT Corporation, Atsugi, Japan; ^2^Open Innovation Institute, Kyoto University, Kyoto, Japan; ^3^Faculty of Health Science, Health Science University, Fujikawaguchiko, Japan; ^4^Division of Cognitive Psychology in Education, Graduate School of Education, Kyoto University, Kyoto, Japan; ^5^BKC Research Organization of Social Sciences, Ritsumeikan University, Kusatsu, Japan; ^6^Yoshika Institute of Psychology, Kanoashi, Japan

**Keywords:** focused attention meditation, open monitoring meditation, cortisol, respiration, autonomic cardiac modulation

## Abstract

Mindfulness-based interventions (MBIs) have been used widely as a useful tool for the alleviation of various stress-related symptoms. However, the effects of MBIs on stress-related physiological activity have not yet been ascertained. MBIs primarily consist of focused-attention (FA) and open-monitoring (OM) meditation. Since differing effects of FA and OM meditation on brain activities and cognitive tasks have been mentioned, we hypothesized that FA and OM meditation have also differing effects on stress-related physiological activity. In this study, we examined the effects of FA and OM meditation on autonomic cardiac modulation and cortisol secretion. Forty-one healthy adults (aged 20–46 years) who were meditation novices experienced 30-min FA and OM meditation tasks by listening to instructions. During resting- and meditation-states, electrocardiogram transducers were attached to participants to measure the R-R interval, which were used to evaluate heart rate (HR) and perform heart rate variability (HRV) analyses. Saliva samples were obtained from participants pre- and post-meditation to measure salivary cortisol levels. Results showed that FA meditation induced a decrease in HR and an increase in the root mean square of successive differences (rMSDD). In contrast, OM meditation induced an increase in the standard deviation of the normal-to-normal interval (SDNN) to rMSSD ratio (SDNN/rMSSD) and a decrease in salivary cortisol levels. These results suggest that FA meditation elevates physiological relaxation, whereas OM meditation elevates physiological arousal and reduces stress.

## Introduction

Mindfulness is the awareness that arises from paying attention to the present moment with intention, without judgment (Kabat-Zinn, 2017). Since the introduction of Mindfulness Based Stress Reduction (MBSR) by Kabat-Zinn (Kabat-Zinn, 1982), a large body of studies have reported that mindfulness meditation and mindfulness-based interventions (MBIs), which typically consist of focused-attention (FA) and open-monitoring (OM) meditation, can induce beneficial effects on various stress-related symptoms. For example, mindfulness meditation can reduce symptoms of depression, anxiety, and pain (Goyal et al., 2014), and has shown to have a significant effect on preventing recurrence of depression (Kuyken et al., 2016). However, a few studies have reported adverse effects of mindfulness meditation and MBIs, such as relaxation-induced anxiety, panic, or re-experiencing of traumatic memories (Dobkin et al., 2012; Van Dam et al., 2018). To understand the mechanisms underlying the positive and negative effects of mindfulness meditation, it is crucial to determine how mindfulness meditation modulates physiological activity, particularly autonomic nerve activity, respiration and cortisol secretion because these have been consistently linked to stress-related symptoms (Grossman, 1983; McEwen, 1998, 2008). Although numerous studies have reported the effects of mindfulness meditation and MBIs on autonomic nerve activity and cortisol secretion, they remain poorly understood and require further exploration.

Several previous studies have reported a greater reduction of respiration rate during meditation compared with during quiet sitting (Lehrer et al., 1999; Cysarz and Büssing, 2005; Ditto et al., 2006). In addition, respiration rate during meditation was reported to be reduced after 8 weeks of MBI training compared with before training (Farb et al., 2013). In addition, the basal respiration rate, measured at rest while not performing any tasks, is reported to be negatively correlated with time spent in intensive retreat practice (Wielgosz et al., 2016). These studies suggest that mindfulness meditation has the effect of reducing respiration rate. However, previous studies in which heart rate variability (HRV) was calculated from heart rate (HR) time series data to estimate cardiac control by the autonomic nerves have reported a variety of results. The high-frequency (HF) component, and the low-frequency (LF) to HF ratio (LF/HF) of HRV have been used in many studies to estimate the strength of cardiac parasympathetic and sympathetic regulation, respectively (Task Force, 1996; Berntson et al., 1997). One study reported that a 4-week MBI increased the HF component of HRV (Ditto et al., 2006). However, other studies reported that the HF component in spontaneous breathing condition was not changed after a 4-week (Park et al., 2018) or 8-week MBI (Nijjar et al., 2014). Concerning LF/HF, one study reported that a 4-week MBI induced a trend toward a decrease in LF/HF (Park et al., 2018). In contrast, other studies demonstrated no reduction in LF/HF after a 6-week (Oken et al., 2017) or 8-week MBI (Joo et al., 2010). One previous study of short-term meditation demonstrated a greater increase in LF/HF in participants performing the OM meditation period of Vipassana meditation (a combination of 10-min FA, 15-min OM, and 5-min loving kindness meditation) compared with participants performing random thinking (Delgado-Pastor et al., 2013). These studies suggest that the effects of MBIs are inconsistent, including both parasympathetic and sympathetic dominance, or the lack of a significant change in parasympathetic or sympathetic cardiac modulation.

Salivary or serum cortisol levels have also been analyzed to investigate the effect of mindfulness meditation and MBIs on cortisol secretion. Medical students who participated in 4 days of MBI showed a decrease in serum cortisol level at eight in the morning (Turakitwanakan et al., 2013). Another study in healthy participants similarly reported a trend decrease in salivary cortisol levels measured after awakening, following 8 weeks of an MBSR program (Jensen et al., 2012). In contrast, another study found no effect of a 6-week MBSR program on average daily salivary cortisol levels (Klatt et al., 2009).

Taken together, although the effects of mindfulness meditation and MBIs on autonomic activity, respiration and cortisol secretion have been evaluated using similar physiological indices, the results of previous studies of autonomic activity and cortisol secretion remain controversial. To determine the effects of mindfulness meditation and MBIs on physiological activity, we focused on the differences in the proportion of FA and OM meditation in MBIs (Lutz et al., 2008). FA meditation entails the voluntary focusing of attention on a chosen object (Lutz et al., 2008). Having a chosen object enables meditators to increase top-down selective attention regulation and more easily maintain attention even with the presence of distractors (Hasenkamp et al., 2012; Colzato et al., 2015a; Fujino et al., 2018). For emotion regulation during FA meditation, focused attention reduces sensitivity to emotional distractors that are presented externally to the focus of attention (Desbordes et al., 2014; Guendelman et al., 2017). For example, FA meditation reduces both pain intensity and pain unpleasantness ratings evoked by noxious stimulation external to the focus of attention (Zeidan et al., 2011).

OM meditation involves non-reactive moment-to-moment monitoring of the content of one’s experiences (Lutz et al., 2008). Nonreactive monitoring without a chosen object enables meditators to weaken top-down selective attention regulation and perceive contents of experiences as simply objects of awareness, rather than as distractors (Colzato et al., 2015a; Fujino et al., 2018), which defines the state of mindfulness. For emotion regulation during OM meditation, meditators monitor emotional stimuli with an attitude of acceptance; every momentary experience is accepted as it is, instead of suppressing or avoiding the emotion. This mindfulness state does not reduce pain intensity evoked by electric stimuli; however, it does reduce pain unpleasantness ratings (Gard et al., 2012).

Although there are differences in attention and emotion regulation techniques between FA and OM meditation, to our knowledge, there have not been any studies that have compared autonomic activity during FA and OM meditation with those during resting state, and cortisol secretion before FA and OM meditation and after FA and OM meditation. Given that Buddhist tradition describes FA meditation as having a calming effect on the mind, whereas OM meditation is active and energy gathering (Brown and Ryan, 2004), there may be differences in the effects of FA and OM meditation. To support this notion, a previous study that examined the subjective feelings of effort and HR during meditation, without a comparison with resting state, suggested that OM meditation requires cognitive resources and induces stronger physiological arousal compared with FA meditation (Lumma et al., 2015). On the basis of these findings, we expect FA meditation to increase parasympathetic nerve activity and OM meditation to increase sympathetic nerve activity. Furthermore, because OM meditation induces a state of mindfulness that is related to stress reduction, OM meditation would likely reduce cortisol levels. FA meditation may also reduce cortisol levels; previous research in other fields have demonstrated an increase in relaxation levels alongside a decrease in cortisol levels (Phillips et al., 2008; Handlin et al., 2011).

To investigate the different effects of FA and OM meditation, we focused on short-term FA and OM meditation interventions in meditation-naive participants. Several recent studies in psychological regions applied short-term FA and OM meditation interventions in meditation-naive participants and revealed varied effects on the psychological processes of FA and OM meditation. Studies by Colzato et al. showed differences in cognitive control following 17 min each of FA and OM meditation in meditation-naive participants (Colzato et al., 2015a,b). These studies suggest that short-term meditation tasks in meditation-naive participants can be valuable for disentangling the effects of FA and OM meditation on autonomic activity and cortisol secretion. In this study, we used a guided 30-min meditation that we developed (Fujino et al., 2019) in which step-by-step instructions for FA and OM meditation are provided verbally to participants.

The purpose of this study was to identify the effects of FA and OM meditation on sympathetic and parasympathetic nerve activity and cortisol secretion. We measured HR and calculated HRV. We hypothesized that FA meditation would facilitate parasympathetic cardiac modulation, whereas OM meditation would facilitate sympathetic cardiac modulation. Moreover, we investigated whether OM and FA meditation decrease cortisol levels. The results enable the identification of a possible relationship between the physiological and psychological mechanisms of FA and OM meditation.

## Materials and Methods

### Ethics Statement

Before the experiment, participants were provided with an information sheet that outlined the general purpose of the study and informed them that they could withdraw from the study at any time without penalty. All participants provided informed consent. The study was approved by the Ethics and Safety Committees of NTT Communication Science Laboratories and was conducted in accordance with the Declaration of Helsinki.

### Participants

We recruited 53 people with no history of diabetes, hypertension or thyroid dysfunction, who did not receive any medication treatment. After excluding one male participant who did not attend the study session, 52 participants (25 men) aged 20–50 years [mean age ± standard deviation (SD), men: 27.2 ± 8.9 years; women: 32.6 ± 8.7 years] participated in the study. On the experimental day, we checked whether participants felt ill, to avoid collecting data from unhealthy participants. All participants had normal hearing and no experience with meditation. The participants were informed that they will undergo a mental exercise and will be listening to recorded instructions played on a loudspeaker, which they will follow to perform the exercise. To avoid the participants interpreting the study as a religious exercise, we refrained from using the term “meditation” throughout the experiment. Participants were asked to provide saliva samples before and after the mental exercise. Participants were paid for their participation.

### Instructions of Meditation

We used 30-min Japanese instructions for FA and OM meditation that were developed for a previous study (Fujino et al., 2019), which were spoken by a highly experienced meditation instructor. The instructions for FA meditation were developed on the basis of a previous study (Colzato et al., 2012), where some of the words, such as non-judgment, were removed because they were related to OM meditation. The OM meditation instructions were developed on the basis of the manual of Anapanasati Sutta (Inoue, 2005) in accordance with the structure of FA meditation instructions. The structure of both instructions consisted of five parts: “overview of instruction,” “how to assume the correct posture,” “how to breathe,” “how to perform the mental exercise,” and “how to finish.” Each part consisted of alternations of the audio instruction and mental practice phases.

During FA meditation, participants were asked to allow the breath to arise naturally and to pay attention to the breath. For the first step of FA meditation, participants were instructed to pay attention to their breath by counting each breath from one to ten for several minutes. For the second step, they were instructed to pay attention to the breath while thinking “breathe in” and “breathe out” to themselves for several minutes. For the third step, participants were instructed to simply pay attention to the breath for several minutes without verbally labeling each breath. Additionally, participants learned how to deal with mind wandering; when they noticed that their mind had become distracted, they were instructed to be aware of the distraction without thinking about the details, and to say “distraction” in their mind and return their attention back to the breath as soon as possible. In the final 5 min, they were asked to pay attention to their natural breath without verbal labeling and to return to the breath without the verbal cue, whenever they noticed their mind wandering. By following this process of FA meditation, participants learned how to gradually increase top-down selective attention regulation by counting their breath using numbers or words, rather than be aware of moment-to-moment sensory experiences.

For OM meditation, participants were instructed to allow their breath to arise naturally and be aware of sensations as they occurred. For the first step, participants were instructed to be aware of the sensation of the breath around their nostrils for several minutes. For the second step, they were instructed to be aware of their experiences of hearing, olfaction, gustation, or somatosensation without judgment, for several minutes per sensation. The participants also learned how to deal with mind wandering. Whenever participants noticed that their mind had become distracted, they were instructed to be aware of the distraction without judgment or criticism of themselves and to allow time to feel the impact of their thoughts and emotions on their body for several minutes. For the final step, participants were instructed to be aware of the sensation of the breath around their nostrils, feel the impact of their thoughts and emotions on their body whenever their mind wandered, and return to their breath slowly and gently for 5 min. During OM meditation, participants gradually learned how to be aware of moment-to-moment sensory experience as they are.

### Physiological Measures

#### Salivary Cortisol

**FIGURE 1:**
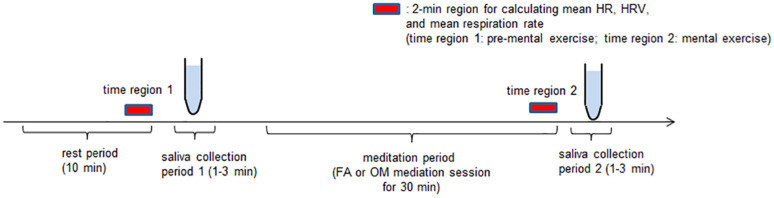
Experimental paradigm. Participants experienced one 10-min silent period as a rest period, two saliva collection periods (1 and 2), each of which were 1–3 min long, and one 30-min mental exercise period. After the rest period, in saliva collection period 1, we took a saliva sample of at least 1 mL from the participants for a cortisol assay. For the next mental exercise period, which lasted 30 min, FA or OM meditation instructions were presented. Finally, the participants provided saliva during saliva collection period 2. The participants performed both FA and OM meditation tasks on the same day.

In contrast to collecting blood samples, obtaining saliva samples is non-invasive and includes protein-free cortisol that accurately reflects physiological processes that cannot be confirmed by total blood hormone levels (Hofman, 2001; Goncharov et al., 2006). Therefore, we used participants’ saliva to measure cortisol levels. Saliva samples (1 mL/collection) were collected in cold conical tubes directly from each participant via passive drool. Saliva collection was performed twice for each meditation experiment; immediately before starting the meditation session (saliva collection period 1) and immediately after the end of the meditation session (saliva collection period 2) (Figure 1). All samples were frozen immediately and stored at −80°C until they were required for measurement.

The cortisol concentration in the saliva samples was determined using a liquid chromatography-tandem mass spectrometry system. All analyses were performed using the standard protocols of ASKA Pharma Medical Co., Ltd. (Fujisawa, Japan) (Matsui et al., 2009; Yamashita et al., 2009), which is well established for various types of steroid hormonal assays. The staff at the company were not informed about the sample content or the nature of the study.

#### Heart Rate and Respiration Rate

Electrocardiograms (ECGs) and an elastic chest band Polyam-RESP (Nihon Santeku, Japan) were used to measure interbeat intervals (R-R intervals) and respiration, respectively, throughout the experiment. Analog data were amplified and digitized using a BIOPAC MP150 (BIOPAC Systems, United States). The sampling rate was 1,000 Hz for both the ECG and respiration measurements.

To calculate the R-R intervals of the ECGs, R-wave detection was performed using the AcqKnowledge analysis software (BIOPAC MP150, United States). The data were visually screened to eliminate any inappropriate R-wave detection that may be attributed to artifacts, such as from movement. For HR analysis, the screened R-R interval data were resampled at 10 Hz using cubic spline interpolation, and the interpolated R-R interval data were converted into second-by-second values and expressed as beats per minute (BPM) by dividing 60 by each value.

To calculate respiration rate, raw respiration signals were filtered using a 0.05–0.5 Hz bandpass filter. Peak detection for the filtered respiration signals was performed using AcqKnowledge to obtain peak-to-peak intervals. The peak-to-peak interval data were then resampled at 10 Hz using linear interpolation. The interpolated data were converted into second-by-second values and expressed as cycles per minute (CPM) by dividing 60 by each value.

#### Heart Rate Variability

We performed two types of HRV analyses. The first one was frequency-domain HRV, which is similar to that reported in a previous study (Ooishi et al., 2017). A fast Fourier transformation (FFT) was applied to the interpolated R-R interval data after removing linear trend to calculate the HRV power spectra using a Hanning window. LF and HF components were obtained by integrating the power spectra over their respective ranges of 0.04–0.15 Hz and 0.15–0.50 Hz. The peak HRV associated with respiration [respiratory sinus arrhythmia (RSA)] is regarded as an index of cardiac parasympathetic modulation (Katona and Jih, 1975; Porges, 1986). Because this peak is commonly involved in the HF components, the magnitude of the HF component is thought to correspond to the strength of cardiac parasympathetic modulation (Berntson et al., 1997). LF/HF corresponds to cardiac sympathovagal balance (Malliani et al., 1994; Montano et al., 1994; Task Force, 1996). The magnitude of the LF component involves both cardiac parasympathetic and sympathetic cardiac control (Pomeranz et al., 1985). An FFT was applied to each 2-min window of the interpolated 10-Hz-data series of R-R intervals. The magnitude of each spectral component was evaluated using the natural logarithms of a power (lnLF and lnHF). LF/HF was evaluated using the natural logarithm of LF/HF (ln[LF/HF]).

The second analysis was the time-domain HRV, which was used to evaluate two variables: the standard deviation of the normal-to-normal (SDNN) R-R interval and the root mean square of the successive differences (rMSSD) of the R-R interval (Task Force, 1996). The strength of the cardiac parasympathetic control of the heart can be estimated by the magnitude of rMSSD that is robust against changes in respiration rate (Penttilä et al., 2001). The ratio of SDNN to rMSSD (SDNN/rMSSD) was used as an index of cardiac sympathovagal balance (Waring et al., 2008; Kang et al., 2016). The analyses of the SDNN and rMSSD were performed for each 2-min window of the raw R-R intervals.

The application of HRV analyses was performed twice for each meditation experiment. The first application was performed on the interpolated R-R data in the last 2 min during the 10-min rest period before saliva collection period 1 (time region 1), and the second was performed in the last 2 min of the final 5-min period of silence in the 30-min meditation session (time region 2) (Figure 1). Data indicating inappropriate changes in HR due to artifacts, such as coughing or movement, within the last 5-min in the rest period before meditation and the period of silence during meditation were excluded from the analysis. If there was a period of at least 2 min of data collection before the excluded event, we selected this last 2 min immediately before the excluded event as time region 1 or 2. If there was insufficient data length before the excluded event, we prolonged the measurement until we were able to acquire at least 2 min of stable data to determine time region 1 or 2.

Within time regions 1 and 2, the mean values of HR and respiration rates (mean HR, mean respiration rate, respectively) were taken to perform statistical evaluation noted below.

#### Design and Experimental Procedure

We used a 2 × 2 within-subjects design, where time (pre-mental exercise vs. mental exercise for HRV indices, mean HR, and mean respiration rate; pre-mental exercise vs. post-mental exercise for salivary cortisol level) and meditation (FA meditation vs. OM meditation) were the independent variables. The dependent variables were: the indices for the strength of the autonomic cardiac control (lnLF, lnHF, ln[LF/HF], SDNN, rMSSD, SDNN/rMSSD, and mean HR), mean respiration rate, and salivary cortisol level.

This study comprised two meditation sessions. On each experimental day, two participants came to the laboratory and experienced both meditations (FA and OM meditations) alternately. When one participant experienced one of the meditation experiments, another participant waited in a separate resting room. The interval between the two meditation sessions was at least 2 h for each participant. The order of the meditation sessions was completely randomized. The experiments were performed in a sound-insulated room and participants sat in a chair. All experiments were conducted between 13:00 and 18:00 to control for diurnal variation in hormone levels. The participants were instructed to not drink anything containing alcohol or caffeine after 20:00 the previous day and to not consume anything except for still water after lunch (11:30–12:00) on the day of the experiment. On the day of the experiment, participants received general information about the experiment and written consent was obtained. The experimental procedure consisted of four periods: rest period, saliva collection period 1, meditation period, and saliva collection period 2. Prior to the meditation session, the participants sat in a chair and were attached to ECG transducer electrodes for 10 min to allow them to become familiarized with the experimental environment; this was the rest period. We then collected saliva samples of at least 1 mL from the participants in saliva collection period 1. This saliva sample was used as the level of salivary cortisol during the “pre-mental exercise.” When the meditation session started (meditation period), participants were presented with the voice instructions of either the FA or OM meditation for 30 min. Finally, a second saliva sample was obtained from participants in saliva collection period 2, which was used as the salivary cortisol level during the post-mental exercise.

#### Data Analysis

Data of several participants were excluded from the analyses. One woman became unwell during the experiment and withdrew from the study. ECGs of three women could not be measured because the electrodes became detached. Cortisol levels before meditation in two men were excluded following the Smirnov-Grubbs test, which showed excessive cortisol levels compared with other participants. Moreover, the HRs before meditation of these participants were too high (i.e., 98.2 and 113.5 bpm, respectively, before FA meditation); therefore, all data of both these participants were excluded. A normal R wave could not be detected in the ECG data of one male participant, which prompted us to stop the ECG recording. Because of an electrical problem, saliva samples of two men and two women that were stored in the freezer were thawed and were warmed to room temperature for at least 1 day, so these samples were excluded from the analysis. In total, complete data were collected from 41 participants (20 men: 25.8 ± 1.6 years; 21 women: 30.7 ± 2.0 years). All statistical analyses were conducted on the data of these 41 participants.

Data are presented as means ± *SD*, and *p* < 0.05 was considered statistically significant. Before applying two-factor repeated-measures analyses of variance (ANOVAs), we performed paired *t*-tests between the values at pre-mental exercise of FA meditation and OM meditation to examine whether or not there was a significant difference or trend toward a difference between FA and OM meditation at pre-mental exercise for each parameter. We analyzed the influence of meditation on cortisol secretion in saliva, mean HR, HRV indices and mean respiration rate using separate two-factor repeated-measures ANOVAs with meditation (FA meditation vs. OM meditation) and time (pre-mental exercise vs. mental exercise for HRV indices, mean HR and mean respiration rate; pre-mental exercise vs. post-mental exercise for salivary cortisol level) as within-subjects factors. We used Pearson’s correlations to examine the associations between the ratio of change (post-mental exercise divided by pre-mental exercise for changes in cortisol level, mental exercise divided by pre-mental exercise for changes in autonomic modulations) in cortisol level with that of each parameter of autonomic modulation. When taking the ratio of the changes in the power of LF component, HF component, and LF/HF, the natural logarithm was not used.

To evaluate how frequently the respiration rate entered the LF component region (0.04–0.15 Hz), we calculated the total time during which the respiration rates in time regions 1 and 2 were below 9 CPM. Two-factor repeated-measures ANOVA was applied with meditation (FA meditation and OM meditation) and time (pre-mental exercise and mental exercise) as within-subjects factors.

After applying ANOVA, simple main effects tests were performed only when the ANOVA revealed a significant meditation × time interaction, or a trend toward a meditation × time interaction.

We performed a sphericity test (Mendoza test) and applied Huynh–Feld corrections when we detected sphericity violations.

All statistical analyses were performed using JMP 12.2 (SAS Institute Inc., United States).

## Results

### Effect of FA and OM Meditation on Cardiac Autonomic Modulation and Respiration

Before conducting ANOVAs, we examined whether or not there was a significant difference, or trend toward a difference, between FA and OM meditation at pre-mental exercise for each parameter. We found that there were no significant differences and no trends toward a difference between pre-mental exercise of FA and OM meditation for any parameters. All of the mean values for each parameter and the evaluation of the similarity with paired *t*-tests are summarized in Table 1.

**TABLE 1:** Summary of the mean value (SD) of each measure of each condition (*n* = 41).

	**FA meditation**		**OM meditation**		***P*-value**
**Measures**	**Pre-mental**	**Mental**	**Pre-mental**	**Mental**	**FA vs. OM meditation**
	**exercise**	**exercise**	**exercise**	**exercise**	**at pre-mental exercise**
mean HR (BPM)	74.0 (10.3)	71.4 (10.3)	73.9 (8.8)	73.8 (9.8)	0.94
ln LF (ln-ms^2^)	6.01 (1.29)	6.41 (1.04)	5.97 (1.06)	6.21 (1.00)	0.79
ln HF (ln-ms^2^)	5.42 (1.36)	5.59 (1.17)	5.39 (1.03)	5.30 (1.11)	0.79
ln(LF/HF)	0.59 (1.28)	0.82 (0.89)	0.58 (1.00)	0.91 (1.03)	1.00
SDNN (ms)	57.9 (27.2)	59.7 (26.6)	54.1 (23.2)	58.1 (25.1)	0.18
rMSSD (ms)	36.8 (20.1)	41.1 (23.8)	34.5 (15.0)	33.7 (15.7)	0.26
SDNN/rMSSD	1.72 (0.66)	1.65 (0.54)	1.66 (0.51)	1.85 (0.56)	0.53
Mean respiration rate (CPM)	15.6 (2.9)	14.0 (3.5)	15.4 (2.7)	14.4 (3.1)	0.69

	**FA meditation**		**OM meditation**		***P*-value**
**Measures**	**Pre-mental**	**Post-mental**	**Pre-mental**	**Post-mental**	**FA vs. OM meditation**
	**exercise**	**exercise**	**exercise**	**exercise**	**at pre-mental exercise**

Cortisol level (pg/mL)	803 (462)	781 (615)	947 (577)	734 (400)	0.16

**FIGURE 2:**
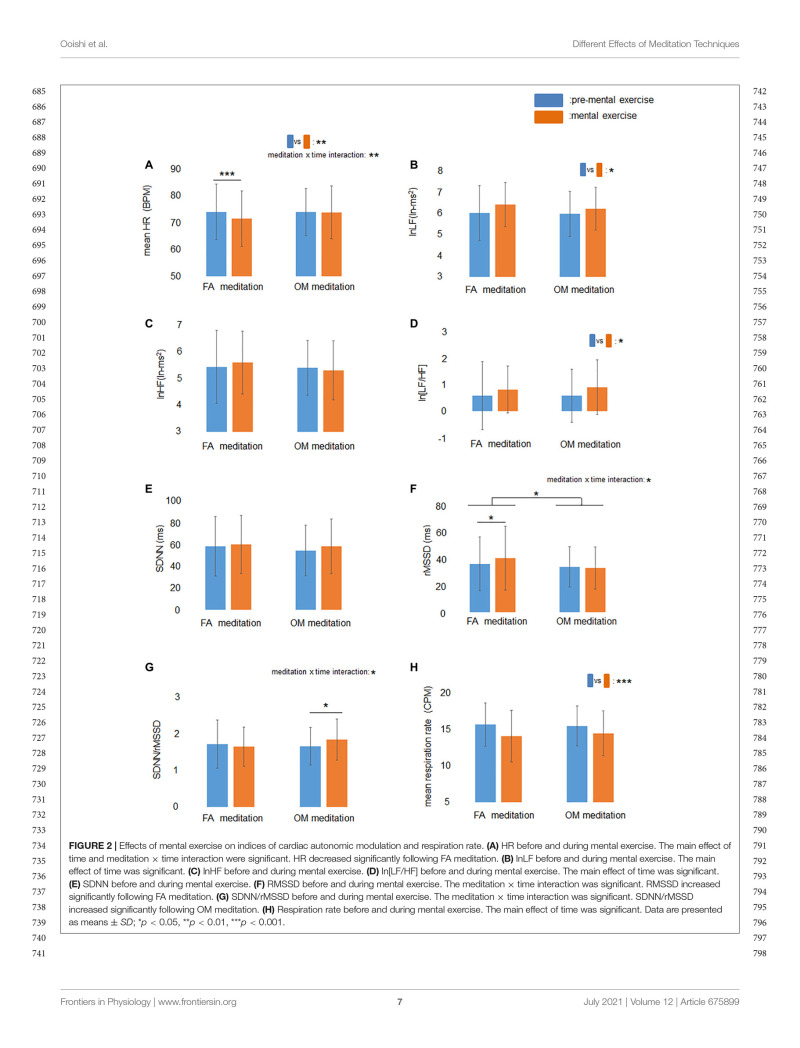
Effects of mental exercise on indices of cardiac autonomic modulation and respiration rate. **(A)** HR before and during mental exercise. The main effect of time and meditation × time interaction were significant. HR decreased significantly following FA meditation. **(B)** lnLF before and during mental exercise. The main effect of time was significant. **(C)** lnHF before and during mental exercise. **(D)** ln[LF/HF] before and during mental exercise. The main effect of time was significant. **(E)** SDNN before and during mental exercise. **(F)** RMSSD before and during mental exercise. The meditation × time interaction was significant. RMSSD increased significantly following FA meditation. **(G)** SDNN/rMSSD before and during mental exercise. The meditation × time interaction was significant. SDNN/rMSSD increased significantly following OM meditation. **(H)** Respiration rate before and during mental exercise. The main effect of time was significant. Data are presented as means ± *SD*; *p < 0.05, **p < 0.01, ***p < 0.001.

The ANOVA for mean HR revealed a significant main effect of time (*p* = 0.0095) and a meditation × time interaction (*p* = 0.0042; Figure 2A). The ANOVA for the factors of frequency-domain HRV revealed a significant main effect of time (*p* = 0.036) for lnLF (Figure 2B), no significant main effect or meditation × time interaction for lnHF (Figure 2C), and a significant main effect of time (*p* = 0.026) for ln[LF/HF] (Figure 2D). The ANOVA for the factors of time-domain HRV revealed no significant main effect or meditation × time interaction for SDNN (Figure 2E), a significant main effect of meditation (*p* = 0.018) and a meditation × time interaction (*p* = 0.048) for rMSSD (Figure 2F), and a significant meditation × time interaction (*p* = 0.035) for SDNN/rMSSD (Figure 2G).

Simple main effects tests demonstrated that rMSSD during FA meditation (41.1 ms) was significantly higher than before FA meditation (36.8 ms) (*p* = 0.030), and mean HR during FA meditation (71.4 BPM) was significantly lower than before FA meditation (74.0 BPM) (*p* = 0.0001). There was no significant difference between SDNN/rMSSD during FA mediation (1.65) and before FA meditation (1.72) (*p* = 0.41). A significant increase in SDNN/rMSSD (*p* = 0.033) was observed after OM meditation (1.84) compared with before OM meditation (1.66), whereas there were no significant differences between rMSSD during OM mediation (33.7 ms) and before OM meditation (34.5 ms) (*p* = 0.67), or between HR during OM mediation (73.8 BPM) and before OM meditation (73.9 BPM) (*p* = 0.85).

The ANOVA for mean respiration rate revealed a significant main effect of time (*p* < 0.0001; Figure 2H), indicating that respiration rate during meditation was lower than before meditations.

The results of the ANOVAs (main effects and interactions) and simple main effects tests are summarized in Table 2.

**TABLE 2:** Summary of the main effects, meditation × time interaction and the simple main effects tests of the two-factor repeated measures ANOVA (*n* = 41).

	**Main effect of meditation**			**Main effect of time**			**Meditation** × time interaction			**Simple main effect test (pre- vs. mental exercise)**		
**Measures**	***F*(1, 40)**	***P*-value**	**Partial**	***F*(1, 40)**	***P*-value**	**Partial**	***F*(1, 40)**	***P*-value**	**Partial**	**Meditation**	***F*(1, 80)**	***P*-value**
	**value**		**η^2^**	**value**		**η^2^**	**value**		**η^2^**		**value**	
HR (BPM)	1.81	0.19	0.043	7.42	0.0095**	0.16	9.21	0.0042**	0.19	FA meditation	16.2	0.0001***
										OM meditation	0.0383	0.85
lnLF (ln-ms^2^)	1.03	0.32	0.025	5.35	0.026*	0.12	0.600	0.44	0.015	FA meditation	–	–
										OM meditation	–	–
lnHF (ln-ms^2^)	2.59	0.12	0.061	0.167	0.69	0.004	2.43	0.13	0.057	FA meditation	–	–
										OM meditation	–	–
ln(LF/HF)	0.122	0.73	0.003	4.71	0.036*	0.11	0.170	0.68	0.004	FA meditation	–	–
										OM meditation	–	–
SDNN (ms)	1.31	0.26	0.032	1.23	0.27	0.030	0.23	0.63	0.006	FA meditation	–	–
										OM meditation	–	–
rMSSD (ms)	6.10	0.018*	0.13	1.37	0.25	0.033	4.17	0.048*	0.094	FA meditation	4.90	0.030*
										OM meditation	0.180	0.67
SDNN/rMSSD	1.19	0.28	0.029	0.849	0.36	0.021	4.76	0.035*	0.11	FA meditation	0.67	0.41
										OM meditation	4.69	0.033*
Respiration rate (CPM)	0.0585	0.81	0.001	18.9	<0.0001***	0.32	0.750	0.39	0.018	FA meditation	–	–
										OM meditation	–	–

	**Main effect of meditation**			**Main effect of time**			**Meditation** × time interaction			**Simple main effect test (pre- vs. post-mental exercise)**		
**Measures**	***F*(1, 40)**	***P*-value**	**Partial**	***F*(1, 40)**	***P*-value**	**Partial**	***F*(1, 40)**	***P*-value**	**Partial**	**Meditation**	***F*(1, 80)**	***P*-value**
	**value**		**η^2^**	**value**		**η^2^**	**value**		**η^2^**		**value**	

Cortisol level (pg/mL)	0.307	0.58	0.008	4.40	0.042*	0.099	4.23	0.046*	0.096	FA meditation	0.0913	0.76
										OM meditation	8.58	0.0045**

Simple main effects tests were performed only when ANOVA showed a significant meditation × time interaction or a trend toward an interaction. *p < 0.05; **p < 0.01; ***p < 0.001.

### Reanalysis of the Effects of FA and OM Meditation on Cardiac Autonomic Modulation and Respiration Without Participants Who Exhibited Mean Respiration Rates Less Than 9 CPM at Least Once

Two participants exhibited mean respiration rates less than 9 CPM at least once in time regions 1 and 2 of FA and OM meditations. Because a respiration rate less than 9 CPM indicates that the RSA peak is involved in the LF component region, it is impossible to clearly distinguish the strength of the cardiac sympathetic and parasympathetic modulation by calculating the frequency-domain HRV. Therefore, we reanalyzed the frequency-domain HRV excluding the data from the corresponding participants.

Before applying ANOVAs, we examined whether or not there was a significant difference or trend toward a difference between FA and OM meditation at pre-mental exercise for each parameter. We found that there was no significant difference or trend toward a difference between pre-mental exercise of FA and OM meditation in any parameters. All of the mean values for each parameter and the evaluation of the similarity with paired t-tests are summarized in Table 3.

**TABLE 3:** Summary of the mean value (SD) of each measure of each condition without the data of participants who showed a mean respiration rate under 9 CPM at least once within time region 1 or 2 in the FA or OM meditation experiment (*n* = 39).

	**FA meditation**		**OM meditation**		***P*-value**
**Measures**	**Pre-mental**	**Mental**	**Pre-mental**	**Mental**	**FA vs. OM meditation**
	**exercise**	**exercise**	**exercise**	**exercise**	**at pre-mental exercise**
Mean HR (BPM)	73.5 (10.4)	70.9 (10.4)	73.9 (9.0)	73.5 (10.0)	0.71
ln LF (ln-ms^2^)	6.09 (1.27)	6.42 (1.06)	6.00 (1.05)	6.20 (1.03)	0.54
ln HF (ln-ms^2^)	5.54 (1.24)	5.68 (1.12)	5.45 (1.00)	5.40 (1.04)	0.52
ln(LF/HF)	0.54 (1.29)	0.74 (0.85)	0.55 (0.96)	0.80 (0.93)	0.98
SDNN (ms)	59.5 (27.0)	60.9 (26.7)	54.9 (23.5)	59.1 (25.2)	0.12
rMSSD (ms)	37.8 (20.0)	42.4 (23.7)	35.3 (14.9)	34.6 (15.5)	0.23
SDNN/rMSSD	1.71 (0.67)	1.62 (0.53)	1.65 (0.52)	1.82 (0.56)	0.48
Mean respiration rate (CPM)	15.6 (3.01)	14.4 (3.28)	15.8 (2.33)	14.6 (3.04)	0.78

	**FA meditation**		**OM meditation**		***P*-value**
**Measures**	**Pre-mental**	**Post-mental**	**Pre-mental**	**Post-mental**	**FA vs. OM meditation**
	**exercise**	**exercise**	**exercise**	**exercise**	**at pre-mental exercise**

Cortisol level (pg/mL)	808 (466)	793 (625)	968 (582)	752 (402)	0.13

**FIGURE 3:**
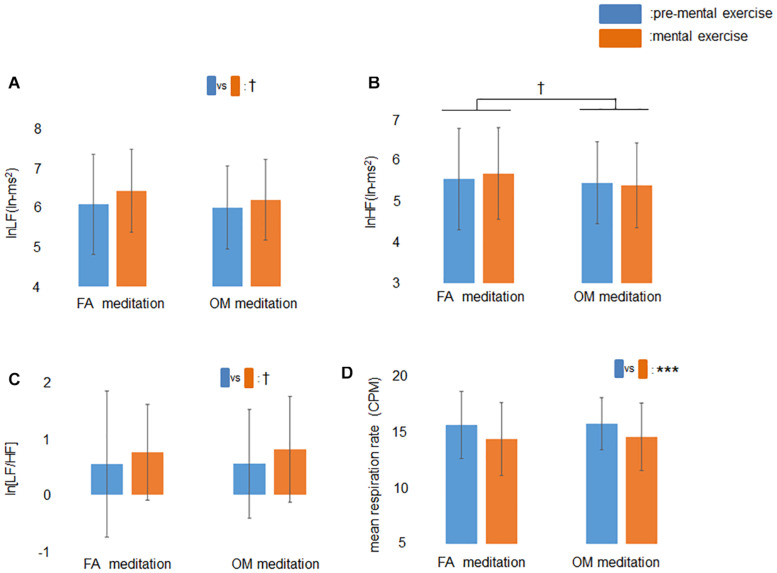
Effects of mental exercise on indices of cardiac autonomic modulation and respiration without those who showed mean respiration rate less than 9 CPM at least once. **(A)** lnLF before and during mental exercise. There was a trend toward a main effect of time. **(B)** lnHF before and during mental exercise. There was a trend toward a main effect of meditation. **(C)** ln[LF/HF] before and during mental exercise. There was a trend toward a main effect of time. **(D)** Respiration rate before and during mental exercise. The main effect of time was significant. Data are presented as means ± *SD*; ^†^*p* < 0.1, ***p < 0.001.

The ANOVA for the factors of frequency-domain HRV revealed a trend toward a main effect of time (*p* = 0.060) for lnLF (Figure 3A), a trend toward a main effect of meditation (*p* = 0.077) for lnHF (Figure 3B), and a trend toward a main effect of time (*p* = 0.071) for ln[LF/HF] (Figure 3C). The ANOVA for mean respiration rate revealed a significant main effect of time (*p* = 0.0003; Figure 3D).

The results of ANOVAs (main effects and interaction) and simple main effects tests are summarized in Table 4.

**TABLE 4:** Summary of the main effects, meditation × time interaction and the simple main effects tests of the two-factor repeated measures ANOVA without the data of participants who showed a mean respiration rate under 9 CPM at least once within time region 1 or 2 in the FA or OM meditation experiments (n = 39).

	**Main effect of meditation**			**Main effect of time**			**Meditation × time interaction**			**Simple main effect test (pre- vs. mental exercise)**		
**Measures**	***F*(1, 38)**	***P*-value**	**Partial**	***F*(1, 38)**	***P*-value**	**Partial**	***F*(1, 38)**	***P*-value**	**Partial**	**Meditation**	***F*(1, 76)**	***P*-value**
	**value**		**η^2^**	**value**		**η^2^**	**value**		**η^2^**		**value**	
HR (BPM)	2.99	0.092^†^	0.073	8.46	0.0060**	0.18	7.57	0.0090**	0.17	FA meditation	15.9	0.0002***
										OM meditation	0.252	0.62
lnLF (ln-ms^2^)	1.75	0.19	0.044	3.77	0.060^†^	0.090	0.497	0.49	0.013	FA meditation	–	–
										OM meditation	–	–
lnHF (ln-ms^2^)	3.30	0.077^†^	0.080	0.201	0.66	0.005	1.42	0.24	0.036	FA meditation	–	–
										OM meditation	–	–
ln(LF/HF)	0.0514	0.82	0.001	3.46	0.071^†^	0.083	0.0505	0.82	0.001	FA meditation	–	–
										OM meditation	–	–
SDNN (ms)	1.63	0.21	0.041	1.12	0.30	0.029	0.356	0.55	0.009	FA meditation	–	–
										OM meditation	–	–
rMSSD (ms)	6.32	0.016*	0.14	1.57	0.22	0.040	3.91	0.055^†^	0.093	FA meditation	4.99	0.028*
										OM meditation	0.103	0.75
SDNN/rMSSD	0.952	0.34	0.024	0.404	0.53	0.011	4.89	0.033*	0.11	FA meditation	1.18	0.28
										OM meditation	3.99	0.049*
Respiration rate (CPM)	0.204	0.65	0.005	15.7	0.0003***	0.29	0.0186	0.89	0.000	FA meditation	–	–
										OM meditation	–	–

	**Main effect of meditation**			**Main effect of time**			**Meditation** × time interaction			**Simple main effect test (pre- vs. post-mental exercise)**		
**Measures**	***F*(1, 38)**	***P*-value**	**Partial**	***F*(1, 38)**	***P*-value**	**Partial**	***F*(1, 38)**	***P*-value**	**Partial**	**Meditation**	***F*(1, 76)**	***P*-value**
	**value**		**η^2^**	**value**		**η^2^**	**value**		**η^2^**		**value**	

Cortisol level (pg/mL)	0.430	0.52	0.011	3.82	0.058^†^	0.091	4.30	0.045*	0.10	FA meditation	0.0362	0.85
										OM meditation	8.00	0.0060**

*Simple main effects tests were performed only when ANOVA showed a significant meditation × time interaction or a trend toward an interaction.*

^†^p < 0.1; *p < 0.05; **p < 0.01; ***p < 0.001.

### Ratio of Time Span of Respiration Rates Under 9 CPM During Rest Period and Mental Exercise

**FIGURE 4:**
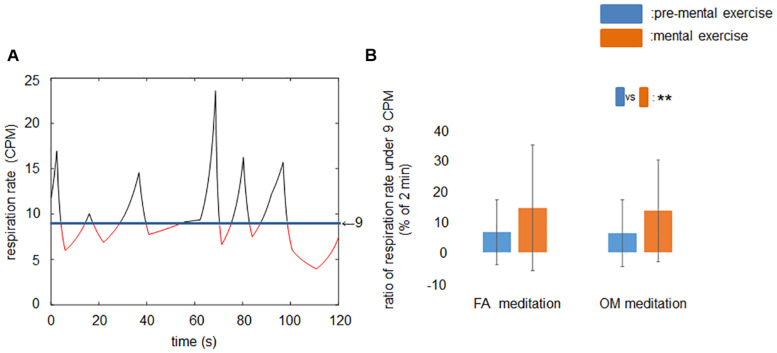
Ratio of time span of respiration rates under 9 CPM during rest period and mental exercise. **(A)** A sample recording of the time series of respiration rate in time region 1 or 2. Red line indicates the respiration rate lower than 9 CPM. **(B)** Ratio of respiration rate under 9 CPM before and during mental exercise. The main effect of time was significant. Data are presented as means ± *SD*; **p < 0.01.

After removing the data of participants who exhibited mean respiration rates less than 9 CPM at least once, however, the frequency-domain HRV indices did not show any significant or trend of meditation × time interaction that could be observed in the time-domain HRV indices. Therefore, we examined the respiration data in time regions 1 and 2 for 39 participants and evaluated how long the respiration rates were below 9 CPM within a 2-min window (Figure 4A).

Before applying the ANOVAs, we examined whether or not there was a significant difference or trend toward a difference between FA and OM meditation at pre-mental exercise condition for each parameter. We found no significant difference or trend toward a difference between pre-mental exercise of FA and OM meditation. All of the mean values and the evaluation of the similarity with paired t-tests are summarized in Table 5.

**TABLE 5:** Summary of the mean value (SD) of ratio of respiration rate under 9 CPM (% of 2 min) in each condition and the similarity between FA and OM meditation in the pre-mental exercise condition (*n* = 39).

	**FA meditation**		**OM meditation**		***P*-value**
**Measures**	**Pre-mental**	**Mental**	**Pre-mental**	**Mental**	**FA vs. OM meditation**
	**exercise**	**exercise**	**exercise**	**exercise**	**at pre-mental exercise**
Ratio of respiration rate under 9 CPM (% of 2 min)	6.3 (11.0)	13.7 (16.8)	6.7 (10.7)	14.6 (20.7)	0.87

The ANOVA revealed a significant main effect of time (*p* = 0.0027; Figure 4B).

### Effect of FA and OM Meditation on Salivary Cortisol Levels

**FIGURE 5:**
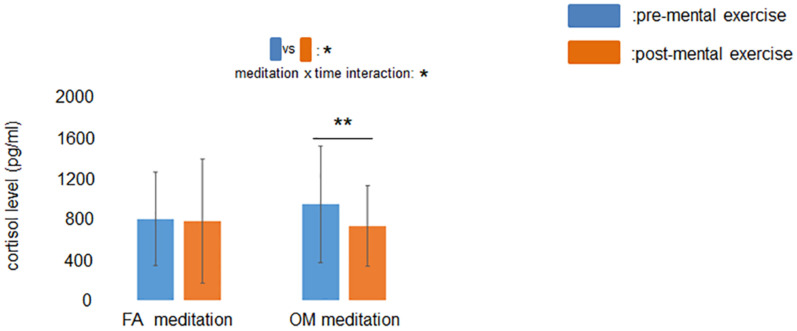
Effect of mental exercise on salivary cortisol levels. The main effect of time and the meditation × time interaction were significant. Salivary cortisol levels were reduced following OM meditation. Data are presented as means ± *SD*; *p < 0.05, **p < 0.01.

The ANOVA revealed a significant main effect of time (*p* = 0.042) and a significant meditation × time interaction (*p* = 0.046) for salivary cortisol level (Figure 5). A simple main effects test demonstrated that cortisol levels after OM meditation (734 pg/mL) were lower than before OM meditation (947 pg/mL) (*p* = 0.0045), whereas there was no significant difference in cortisol level between before (803 pg/mL) and after FA meditation (781 pg/mL) (*p* = 0.76). These results suggested that the salivary cortisol level decreased during OM meditation but not during FA meditation. The mean values and the evaluation of the similarity with paired t-tests are summarized in Table 1. The results of ANOVAs (main effects and interaction) and simple main effects tests are summarized in Table 2.

When we removed the data of individuals who exhibited mean respiration rates less than 9 CPM at least once in time regions 1 and 2 of FA and OM meditations, the cortisol results were not affected (see Tables 3, 4).

### Correlation Between Salivary Cortisol Level Change and Changes in Cardiac Autonomic Modulation

The Pearson’s correlation revealed that there were no significant correlations between the ratio of change in salivary cortisol level and that in any index of cardiac autonomic control (cortisol vs. mean HR: *r* = 0.023; cortisol vs. LF: *r* = 0.037; cortisol vs. HF: *r* = 0.0050; cortisol vs. LF/HF: *r* = 0.050; cortisol vs. SDNN: *r* = 0.12; cortisol vs. rMSSD: *r* = 0.10; cortisol vs. SDNN/rMSSD: *r* = 0.040) (82 data points).

## Discussion

Our study showed that the effects of mindfulness meditation depend on the type of meditation, where FA and OM meditation induced completely different physiological responses. As hypothesized, we confirmed that OM meditation increases cardiac sympathetic modulation, as represented by SDNN/rMSSD, without affecting cardiac parasympathetic modulation, which was represented by rMSSD. In contrast, FA meditation increased cardiac parasympathetic modulation without affecting cardiac sympathetic modulation. In addition, we found that OM meditation reduced salivary cortisol levels, which was not observed following FA meditation. Moreover, we replicated the effect of decreasing respiration rate by meditation as indicated by several previous reports (Lehrer et al., 1999; Cysarz and Büssing, 2005; Ditto et al., 2006).

Frequency-domain HRV has been widely used to evaluate the strength of cardiac autonomic modulation. The cardiac parasympathetic and sympathetic outflow originates from the nucleus ambiguus (NA) (Izzo et al., 1993; Dyavanapalli et al., 2016) and the rostral ventrolateral medulla (RVLM) (Kumagai et al., 2012), respectively. Because the RSA amplitude is well correlated with NA-related cardiac parasympathetic modulation and the RSA peak is usually involved in the range of the HF component of HRV, the power of the HF component is regarded as an index of cardiac parasympathetic modulation (Berntson et al., 1997). However, some previous studies have questioned whether the LF component of HRV is an appropriate index of cardiac sympathetic modulation (Malliani et al., 1991). The LF peak of HRV has been reported to be reduced by at least 50% by either an application of atropine (a muscarinic receptor antagonist) (Houle and Billman, 1999), or selective parasympathectomy (Randall et al., 1991). Another study reported that the LF peak of HRV was largely unaffected by a beta-adrenergic antagonist alone (Reyes del Paso et al., 2013). In addition to an earlier report (Pomeranz et al., 1985), these studies suggest that the LF component of HRV reflects not only sympathetic but also parasympathetic cardiac control.

The LF/HF of HRV may provide an appropriate index for evaluating the strength of cardiac sympathetic modulation. Although LF/HF was traditionally considered to be an index of sympathovagal valance (Malliani et al., 1994; Montano et al., 1994), a direct relationship between LF/HF and the sympathetic cardiac control induced by RVLM neurons has been observed. The microinjection of orexin-A into the RVLM was reported to increase HR and LF/HF (Chen et al., 2013) and depolarize RVLM neurons (Dun et al., 2000). In addition, the microinjection of L-arginine into the RVLM has been reported to facilitate the release of adenosine, resulting in a decrease in HR and LF/HF (Jiang et al., 2011). Moreover, RLVM activity is reported to be suppressed by increasing adenosine release in the RVLM (Thomas and Spyer, 1999). These studies suggest that a change in RVLM activity is consistently correlated with a change in HR and LF/HF. Therefore, the LF/HF seems to reflect cardiac sympathetic modulation originating from the RVLM.

In the context of the previous studies examining frequency-domain HRV discussed above, the current results may suggest that meditation facilitates sympathetic cardiac modulation without any parasympathetic cardiac modulation, because meditation tasks enhanced lnLF and ln[LF/HF] while no change was found in lnHF. However, this interpretation ignores the effect of respiration rate on frequency-domain HRV indices. Meditation tasks have been shown to reduce respiration rate, which presents difficulties in using frequency-domain HRV to evaluate the strength of cardiac autonomic modulation (Lehrer et al., 1999). When the respiration rate reduces, the peak of RSA moves from the HF region (0.15–0.50 Hz) toward the LF region (0.04–0.15 Hz), making it difficult to distinguish the LF region from the RSA component. Because the current results showed a significant decrease in the respiration rate by meditation, there may be an effect of respiration rate on the power of LF or HF region. Time-domain HRV analysis revealed meditation × time interactions, while frequency-domain HRV analysis did not. To remove the effect of the peak of RSA being within the LF region on frequency-domain HRV, we excluded the data of participants who exhibited mean respiration rates under 9 CPM at least once within time regions 1 and 2 in FA and OM meditation experiments, and reanalyzed the frequency-domain HRV. The results, however, revealed that there was still no significant meditation × time interaction, or a trend toward an interaction. We assume that such a difference between the results of frequency-domain HRV and time-domain HRV was derived from the detailed pattern of respiration rate. Therefore, we evaluated the proportion of time the respiration rates were below 9 CPM within a 2-min period, and used the data for statistical evaluation. Although the average proportion was approximately 6.5% in the time region before both FA and OM meditation, the average proportion in the time region during both FA and OM meditation increased to approximately 14%, which was more than twice that before meditation. We suggest that such a fluctuation of the respiration rate during meditation, more frequently falling below 9 CPM compared with before meditation, affected the power of the LF and HF component, resulting in a difficulty to clearly discriminate between the LF component and the RSA component. However, time-domain HRV is less affected by respiratory patterns (Pitzalis et al., 1996; Penttilä et al., 2001). Therefore, we assume that time-domain HRV could reveal significant meditation × time interactions in such a change in the respiration rate, whereas frequency-domain HRV could not. Taken together, we suggest that the varied effects of mindfulness meditation and MBIs on cardiac autonomic modulation in the literature (Ditto et al., 2006; Joo et al., 2010; Delgado-Pastor et al., 2013; Nijjar et al., 2014; Oken et al., 2017; Park et al., 2018) are attributed to the varied proportions of FA and OM meditations in MBIs and the difficulty in discriminating the LF region from the RSA component in frequency-domain HRV, which is mediated by changes in respiration rate.

Contrary to our hypothesis that both FA and OM meditation would reduce cortisol levels, we found that OM meditation exclusively reduced salivary cortisol levels. This result supports the notion that OM meditation induces a state of mindfulness that is related to stress reduction. The effect of mindfulness meditation and MBIs on cortisol secretion varies across studies, where there have been reports of significant decreases (Turakitwanakan et al., 2013), trend decreases (Jensen et al., 2012), and no significant changes (Klatt et al., 2009) in cortisol levels following participation in MBSR programs. On the basis of our results, it appears that the inconsistent results of previous studies are attributed to the difference in the ratio of FA and OM meditation in MBSR programs.

Our results on the relationship between cardiac autonomic modulation and cortisol secretion are inconsistent with earlier studies. It is thought that the activity of the parasympathetic and sympathetic nerves are a physiological index for relaxation (Sakakibara et al., 1994) and fight-flight response (Jansen et al., 1995), respectively, and the level of cortisol in biological tissues, such as saliva, is a biomarker of stress (Hellhammer et al., 2009). The state of relaxation has been shown to be positively related to the anti-stress condition, which should induce a decrease in cortisol levels. Indeed, previous studies have shown decreases in the cortisol levels with relaxation in anti-stress situations (Phillips et al., 2008; Handlin et al., 2011). Furthermore, in an animal study in rats, an application of corticosterone, a glucocorticoid for rodents, was shown to increase the firing rate of cardiovascular neurons in the RVLM (Rong et al., 1999). However, contrasting effects have also been reported in other studies. One study reported a direct relationship between glucocorticoid and sympathetic nerve activity in humans, demonstrating that glucocorticoid agonists reduce sympathetic outflow (Golczynska et al., 1995). Intracerebroventricular administration of a glucocorticoid receptor (GR) antagonist increases blood pressure, which suggests that GR-induced effects of glucocorticoids involve inhibition of cardiac sympathetic modulation (van den Berg et al., 1990). An indirect relationship was also observed in another study that demonstrated that low-tempo music elevates cardiac parasympathetic modulation without changing salivary cortisol levels, whereas high-tempo music reduces salivary cortisol levels and facilitates cardiac sympathetic modulation (Ooishi et al., 2017). Our results are consistent with these adverse effects, whereby OM meditation induced a decrease in cortisol level and an elevation in cardiac sympathetic, but not parasympathetic, modulation.

On the basis of the observed physiological effects of FA and OM meditation, we discuss the possible relationship between the physiological and psychological mechanisms of FA and OM meditation. FA meditation increases top-down selective attention regulation of a chosen object (Lutz et al., 2008). Previous studies have indicated that FA meditation enhances top-down selective attention to stimuli in the external world (Becerra et al., 2017) and thoughts and/or emotions that arise internally (Brefczynski-Lewis et al., 2007). The literature of Buddhist traditions states that this form of concentration can produce highly positive experiences of peacefulness, tranquility, and mental silence (Rahula, 1974). In fact, a previous study reported that brief FA meditation is associated with improvements in relaxation (Kemper and Rao, 2017) and reductions in mind wandering (Brewer et al., 2011). In our study, FA meditation facilitated cardiac parasympathetic modulation, which was in line with previous studies (Takahashi et al., 2005; Phongsuphap et al., 2008). Facilitation of cardiac parasympathetic tone may be an important mechanism that underlies the beneficial effect of the relaxation response (Sakakibara et al., 1994). Our and previous findings suggest that facilitated cardiac parasympathetic modulation induced by FA meditation may play an important role in improving relaxation.

In contrast, OM meditation decreases top-down selective attention regulation without relying on an object and induces an attitude of acceptance to allow each moment to be experienced as it is (Lutz et al., 2008; Desbordes et al., 2014). Brown and Ryan (Brown and Ryan, 2004) stated that OM meditation is active and energy gathering and heightens awareness of the ongoing stream of perceptual phenomena; moreover, this awareness embeds openness to accept all momentary experiences. Furthermore, Lindsay and Creswell (2019) proposed that this kind of acceptance is a key emotion regulation mechanism for the effects of mindfulness interventions on affective, stress, and health outcomes because it allows stressful stimuli to arise and pass without reactivity. In fact, previous research has shown that OM meditation requires cognitive resources, induces stronger physiological arousal compared with FA meditation (Lumma et al., 2015), and also improves the ability to be aware of a painful sensation without experiencing unpleasantness (Perlman et al., 2010; Gard et al., 2012). Furthermore, smartphone-based mindfulness interventions that include awareness and acceptance training suppresses the elevation of cortisol during stressful tasks (Lindsay et al., 2018). In the current study, OM meditation increased the index of cardiac sympathetic modulation, as reported in a previous study showing a greater increase in LF/HF in participants performing OM meditation compared with those performing random thinking (Delgado-Pastor et al., 2013), and reduced salivary cortisol levels. Previous studies have shown that the central sympathetic system enhances arousal (Jones, 2003), and tonic activity of the locus coeruleus, part of the central sympathetic system, induces scanning or labile attention (Aston-Jones et al., 1999). Furthermore, decreased cortisol has been associated with reduced stress (McEwen, 1998). Therefore, both increased sympathetic nerve activity and reduced cortisol levels may underlie the psychological processes of OM meditation despite their seemingly contradictory effects. Furthermore, reduced cortisol levels induced by OM meditation may play an important role in stress reduction.

In conclusion, we found clear differences in the physiological effects between FA and OM meditation. FA meditation facilitated the parasympathetic cardiac modulation without changes in cortisol secretion, whereas OM meditation facilitated the sympathetic cardiac modulation and reduced cortisol secretion. These results suggest that FA meditation has the effect of elevating the state of physiological relaxation, whereas OM meditation elevates physiological arousal and reduces stress. Our findings provide a foundation for determining the appropriate type of MBIs based on the symptoms of patients in clinical settings.

## Limitations

Meditation practitioners often practice FA and OM meditation simultaneously, so differentiating FA and OM meditation effects in an experimental setting is difficult. Therefore, we studied meditation-naïve individuals who participated in FA and OM meditation separately by following step-by-step voice instructions for each type of meditation. We observed significant differences in the effects of FA and OM meditation on physiological response. However, it was not clear whether the facilitated sympathetic cardiac modulation during OM meditation was associated with a fully alert or effortful state during meditation; indeed, previous research has suggested that meditation practitioners gradually reduce the effort required to sustain awareness. In future, it would be valuable to compare the effects of meditation tasks between meditation practitioners and non-meditators.

In addition, the current study did not include an analysis of arterial blood pressure variability (BPV). Because the combination of HRV and BPV analyses offers a more comprehensive approach to evaluate the strength of the sympathetic and parasympathetic cardiac modulation (Parati et al., 1995), it would be valuable for future studies to simultaneously measure ECG and blood pressure, and to evaluate the effects of mindfulness mediation on these measures.

## Data Availability Statement

The raw data supporting the conclusions of this article will be made available by the authors, without undue reservation.

## Ethics Statement

The studies involving human participants were reviewed and approved by the Ethics and Safety Committees of NTT Communication Science Laboratories. The participants provided their written informed consent to participate in this study.

## Author Contributions

YO, MF, and NK designed the physiological experiments. YO collected autonomic response, respiration rate, and cortisol data, analyzed the autonomic response, respiration rate, and cortisol data, and evaluated the results. VI advised on the mindfulness meditation techniques. YO, MF, MN, VI, and NK wrote the manuscript. All authors contributed to the article and approved the submitted version.

## Conflict of Interest

YO and NK were employed by NTT Corporation. The remaining authors declare that the research was conducted in the absence of any commercial or financial relationships that could be construed as a potential conflict of interest.

## Abbreviations

MBSR: Mindfulness Based Stress Reduction
MBI: mindfulness-based intervention
FA: focused attention
OM: open monitoring
HR: heart rate
HRV: heart rate variability
HF: high frequency
LF: low frequency
SDNN: standard deviation of the normal to normal interval
rMSSD: root mean square of the successive differences
ECG: Electrocardiogram
BPV: arterial blood pressure variability.
